# Supplementary material for: The interactome of intact mitochondria by cross-linking mass spectrometry provides evidence for coexisting respiratory supercomplexes
Source: Mol Cell Proteomics. 2017 Dec 8;17(2):216–32. doi: 10.1074/mcp.RA117.000470 (PMC5795388; doi:10.1074/mcp.RA117.000470)
Supplement: Supplemental Data [file supp_RA117.000470_133922_0_supp_23978_fzffwf.zip › spectra_annotation/mito_DR_spectra_annotation/298-1-2-1-6-1.pdf]

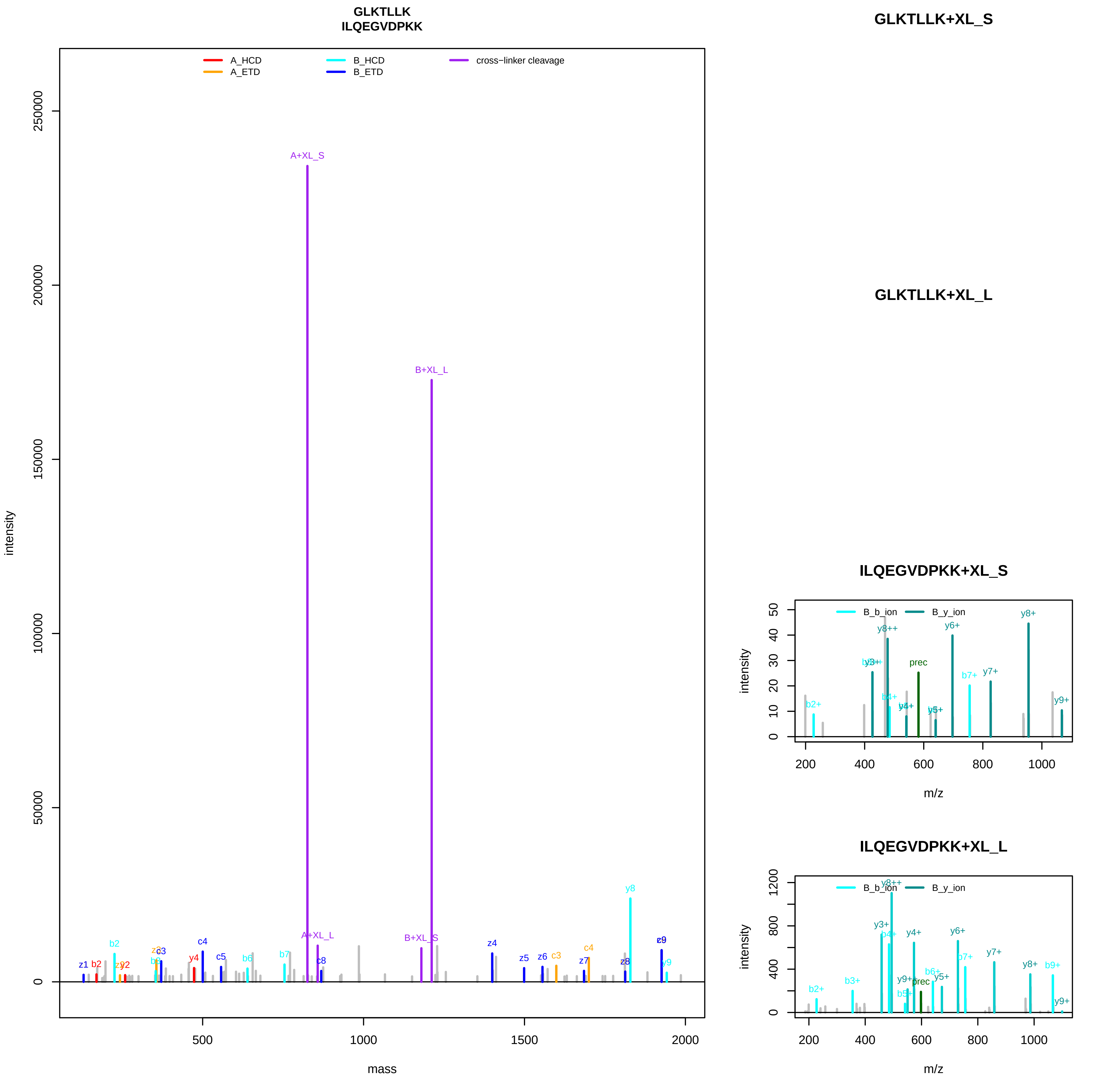

GLKTLLK
ILQEGVDPKK
A_HCD
A_ETD
B_HCD
B_ETD
cross−linker cleavage
A+XL_S
B+XL_L
A+XL_L
B+XL_S
intensity
mass
0
50000
100000
150000
200000
250000
500
1000
1500
2000
z1 b2 y2 c3 c4 c5 b6 b7 c8 y4 b2 z4 z5 z6 c3 z7 c4 z8 y8 z9 y9
GLKTLLK+XL_S
GLKTLLK+XL_L
ILQEGVDPKK+XL_S
B_b_ion
B_y_ion
prec
b2+ y3+ y8++ y6+ y8+ b4+ y4+ y5+ b7+ y7+ y9+
m/z
ILQEGVDPKK+XL_L
y8++ y3+ b4+ y4+ y6+ b7+ y7+ y8+ b9+ b2+ b3+ y9++ b5+ prec b6+ y5+ y9+
